# Dynamic functional network connectivity in patients with a mismatch between white matter hyperintensity and cognitive function

**DOI:** 10.3389/fnagi.2024.1418173

**Published:** 2024-07-17

**Authors:** Siyuan Zeng, Lin Ma, Haixia Mao, Yachen Shi, Min Xu, Qianqian Gao, Chen Kaidong, Mingyu Li, Yuxiao Ding, Yi Ji, Xiaoyun Hu, Wang Feng, Xiangming Fang

**Affiliations:** ^1^Medical Imaging Center, The Affiliated Wuxi People’s Hospital of Nanjing Medical University, Wuxi Medical Center, Nanjing Medical University, Wuxi People’s Hospital, Wuxi, China; ^2^Department of Neurology, The Affiliated Wuxi People’s Hospital of Nanjing Medical University, Wuxi Medical Center, Nanjing Medical University, Wuxi People’s Hospital, Wuxi, China

**Keywords:** cerebral small vessel disease, resting-state functional magnetic resonance imaging, dynamic functional network connectivity, quantitative analysis, anatomy structure

## Abstract

**Objective:**

White matter hyperintensity (WMH) in patients with cerebral small vessel disease (CSVD) is strongly associated with cognitive impairment. However, the severity of WMH does not coincide fully with cognitive impairment. This study aims to explore the differences in the dynamic functional network connectivity (dFNC) of WMH with cognitively matched and mismatched patients, to better understand the underlying mechanisms from a quantitative perspective.

**Methods:**

The resting-state functional magnetic resonance imaging (rs-fMRI) and cognitive function scale assessment of the patients were acquired. Preprocessing of the rs-fMRI data was performed, and this was followed by dFNC analysis to obtain the dFNC metrics. Compared the dFNC and dFNC metrics within different states between mismatch and match group, we analyzed the correlation between dFNC metrics and cognitive function. Finally, to analyze the reasons for the differences between the mismatch and match groups, the CSVD imaging features of each patient were quantified with the assistance of the uAI Discover system.

**Results:**

The 149 CSVD patients included 20 cases of “Type I mismatch,” 51 cases of Type I match, 38 cases of “Type II mismatch,” and 40 cases of “Type II match.” Using dFNC analysis, we found that the fraction time (FT) and mean dwell time (MDT) of State 2 differed significantly between “Type I match” and “Type I mismatch”; the FT of States 1 and 4 differed significantly between “Type II match” and “Type II mismatch.” Correlation analysis revealed that dFNC metrics in CSVD patients correlated with executive function and information processing speed among the various cognitive functions. Through quantitative analysis, we found that the number of perivascular spaces and bilateral medial temporal lobe atrophy (MTA) scores differed significantly between “Type I match” and “Type I mismatch,” while the left MTA score differed between “Type II match” and “Type II mismatch.”

**Conclusion:**

Different mechanisms were implicated in these two types of mismatch: Type I affected higher-order networks, and may be related to the number of perivascular spaces and brain atrophy, whereas Type II affected the primary networks, and may be related to brain atrophy and the years of education.

## 1 Introduction

Cerebral small vessel disease (CSVD) is an intracranial vascular disease involving the microvascular structures of the brain, including the cerebral small arteries, arterioles, capillaries, and small veins (Yang et al., 2022). CSVD is now considered a common cause of vascular cognitive impairment and dementia (Salvadori et al., 2021). CSVD-associated cognitive impairment has been shown to affect a growing proportion of the aging population. However, the mechanisms behind its onset are not fully understood (Ren et al., 2022), and hence there are no effective solutions for its prevention and treatment.

In conventional magnetic resonance imaging (MRI), CSVD is characterized by cerebral white matter hyperintensities (WMHs), recent small subcortical infarcts, lacunes, enlarged perivascular spaces (PVSs), cerebral microbleeds (CMBs), cortical superficial siderosis, and cortical microinfarcts (Wardlaw et al., 2013; Duering et al., 2023), of which WMH is most strongly associated with cognitive impairment (Morrison et al., 2022), contributing to 50% of dementia cases (Wang et al., 2022). However, in clinical practice and in some studies, WMH severity does not fully coincide with cognitive functions in CSVD patients, as demonstrated in cases of mismatch showing mild WMH with cognitive impairment, as well as severe WMH without cognitive impairment (Pasi et al., 2016; Wang et al., 2021), leading to difficulties in the accurate assessment of disease severity in patients clinical settings. Do differences exist in brain function and structure between CSVD patients with and without cognitive impairment under the premise of similar severity of WMH? Are these differences the underlying causes of cognitive impairment?

Advances in functional MRI (fMRI) technology have contributed to the emergence of dynamic functional network connectivity (dFNC) analysis, which allows for a more comprehensive and in-depth imaging evaluation of CSVD patients from a functional perspective, thereby enabling the identification of potential abnormalities beyond conventional imaging features (Xu et al., 2022; Yang et al., 2022; Si et al., 2023; Xie et al., 2023). In some studies, differences in dynamic connectivity between healthy controls and CSVD patients have been observed, as well as between subgroups of CSVD with and without cognitive impairment (Li et al., 2021; Schlemm et al., 2022; Yang et al., 2023). In other studies, they found that the occurrence of mismatch may be associated with certain imaging features of CSVD (Wang et al., 2021; Ye et al., 2022). However, to date, no studies in the literature have directly examined the differences in dFNC between the match and mismatch group; moreover, the anatomical basis for the differences it produces has not been elucidated. Other studies have quantitatively analyzed the macroscopic structure of CSVD and found that there is a certain correlation between cognitive impairment and WMH, perivascular space, brain atrophy, etc. Based on this, we make the hypothesis that the dFNC of patients with WMH cognitive dysfunction has undergone certain changes and is associated with some macroscopic structural changes. Therefore, the aim of this study was to analyze the differences between patients with WMH-cognitive function mismatch and match by dFNC analysis and to explore the mechanisms of its occurrence using quantitative analysis methods.

In summary, this article intends to explore whether there is any abnormality between dFNC and its indicators in CSVD patients, starting from the phenomenon of mismatch between WMH and cognitive function? What is the relationship between these abnormalities and various cognitive domains? And attempt to provide possible explanations for the above abnormal phenomena through quantitative analysis of macroscopic structural changes in CSVD patients, in order to provide more comprehensive and detailed imaging guidance for clinical standardized and scientific diagnosis and treatment strategies for CSVD patients.

## 2 Methods

### 2.1 Patients

A total of 149 CSVD patients were prospectively and consecutively recruited between December 2021 and February 2023. This study has been approved by the Research Ethics Committee of Wuxi People’s Hospital, with approval number KY22080. It was conducted in strict accordance with the Declaration of Helsinki (revised edition). All individuals participating in the study have signed informed consent forms and are aware that their participation is voluntary and they may withdraw at any time without penalty or consequence. All data collected are used for research purposes only and are handled and analyzed with respect to protecting the privacy of the subjects.

The inclusion criteria were as follows: (1) aged 56–75 years; (2) received 6–14 years of education; (3) diagnosed with CSVD according to the 2023 STRIVE-2 diagnostic imaging criteria; (4) able to cooperate with completing the relevant imaging examinations and neuropsychological tests; and (5) voluntary participation and they gave informed consent in writing. The exclusion criteria were as follows: (1) a history of major diseases, including malignant tumors, massive cerebral infarction, brain hemorrhage, intracranial macrovascular diseases, and endocrine diseases (e.g., thyroid and pancreas); (2) systemic autoimmune diseases; (3) severe neurological diseases, such as Alzheimer’s disease, post-cranial surgery, central nervous system infection, primary Parkinson’s disease, traumatic brain injury, epilepsy, brain tumors, etc.; (4) excluding cognitive impairment caused by Alzheimer’s disease and other causes; (5) severe cardiac, pulmonary, and renal insufficiency; (6) hereditary and other rare types of CSVD, such as CADASIL; and (7) contraindications to MRI, such as the presence of metals in the body (e.g., steel pins, plates, dentures, pacemakers, etc.) or claustrophobia.

First, patients were assigned Fazekas scores based on their conventional imaging features, and they were then divided according to the sum of their deep white matter and paraventricular scores (0–6) (Yilmaz et al., 2018) into the mild WMH group (Fazekas scores 1–3) and severe WMH group (scores 4–6). Patients in each group were further subdivided according to their cognitive scale scores: (1) no cognitive impairment group: Mini-Mental State Examination (MMSE) and Montreal Cognitive Assessment (MoCA) scores within the normal range; or (2) cognitive impairment group: MMSE or MoCA scores below the normal range. “Type I mismatch” was defined as mild WMH but with cognitive impairment, and “Type I match” was defined as mild WMH without cognitive impairment. “Type II mismatch” was defined as severe WMH without cognitive impairment, and “Type II match” was defined as severe WMH with cognitive impairment.

### 2.2 Neuropsychological scales

On the day of the MRI scan, the patients completed the relevant neuropsychological scales. The patients were first screened for cognitive impairment using the MMSE and MoCA scales. Then, their cognitive function was assessed using the Auditory Verbal Learning Test (AVLT), Trail Making Test A/B (TMT-A/B), Stroop Color Word Test, Clock Drawing Test (CDT), etc. Among these tests, the recall portion of the AVLT reflects episodic memory function; Stroop C and TMT-B reflect executive function; Stroop A, Stroop B, and TMT-A reflect information processing speed; and CDT reflects visuospatial ability.

### 2.3 Acquisition of imaging data

Image acquisition was performed using a Siemens 3.0 T Prisma MRI scanner (Germany) and a 64-channel coil. During the scan, the heads of the patients were immobilized with foam positioners to minimize head motion artifacts, and the patients were asked to close their eyes but remain awake. Scanning sequences included T2-weighted imaging (T2WI), T2WI-fluid attenuated inversion recovery (FLAIR), susceptibility-weighted imaging (SWI), time-of-flight magnetic resonance angiography (TOF_MRA), three-dimension T1-weighted imaging (3D-T1WI), resting-state fMRI (rs-fMRI), and diffusion-weighted imaging (DWI). The specific parameters were as follows:

1.Conventional sequences: T2WI: TR = 4,960 mm, TE = 109 mm, slice thickness = 5 mm; T2WI-FLAIR: TR = 8,000 ms, TE = 84 ms, slice thickness = 5 mm; SWI: TR = 28 ms, TE = 20 ms, slice thickness = 2.5 mm; TOF_MRA: TR = 21 ms, TE = 3.3 ms, slice thickness = 0.7 mm.
  2.rs-fMRI: gradient-echo echo-planar imaging (GRE-EPI) sequence, TR = 1,500 ms, TE = 31 ms, number of slices = 60, slice thickness = 2.4 mm, slice gap = 0 mm, matrix = 88 × 88, flip angle = 70°, field of view (FOV) = 211 mm × 211 mm, voxel size = 2.4 mm x 2.4 mm x 2.4 mm, scan duration 7 min 40 s.
  3.3D-T1WI: T1-weighted 3D-MPRAGE sequence, TR = 3,000 ms, TE = 2.56 ms, TI = 1,100 ms, flip angle = 7°, number of slices = 208, slice thickness = 0.8 mm, slice gap = 0 mm, matrix 320 × 320, FOV = 256 mm × 256 mm, voxel size = 0.8 mm × 0.8 mm × 0.8 mm, scan duration = 8 min 35 s.
  4.DWI: EPI sequence, TR = 6,800 ms, TE = 75 ms, slice thickness = 1.5 mm, slice gap = 0 mm, matrix = 132 × 128, FOV = 198 mm × 192 mm; diffusion weights were acquired for each slice in 100 gradient directions, with diffusion-weighted coefficient b = 0 s/mm^2^ (10 gradient directions), 1,500 s/mm^2^ (30 gradient directions) and 3,000 s/mm^2^ (60 gradient directions); voxel size = 1.5 mm x 1.5 mm x 1.5 mm, scan duration = 12 min 2 s.
  Three senior radiologists (with over 5 years of experience in diagnostic radiology) were assigned to diagnose the disease and exclude other brain lesions.

## 3 rs-fMRI data processing and analysis

### 3.1 rs-fMRI data preprocessing

The raw rs-fMRI DICOM data of all patients were converted to 4DNIFTI format using dcm2nii (Neuroimaging Informatics Tools and Resources Clearinghouse, United States). Preprocessing of the rs-fMRI data was performed on MATLAB (R2013b, MathWorks, Inc., United States) using the RESTPLUS software (v1.25, China). The specific steps were as follows: (1) removal of the first 10 time points; (2) slice-timing correction, using the middle slice as the reference slice; (3) head-motion correction: removal of patients with head-motion translation >3 mm or rotation >3°; (4) spatial normalization: two-step registration was performed with the help of T1 structural images using the DARTEL normalization strategy; (5) spatial smoothing: smoothing was performed using a 6 mm × 6 mm × 6 mm Gaussian smoothing kernel.

#### 3.1.1 Independent component analysis

ICA was performed on MATLAB using the Infomax algorithm in the GIFT software (v4.0b, Neuroimaging Institute, University of California, Los Angeles, United States), which automatically evaluated the patients to obtain multiple independent components (ICs). The stability of the estimated ICs was then ensured using the ICASSO algorithm. Finally, the GICA algorithm was used to reversely reconstruct the ICs separated at the group level back to each patient. Based on relevant literature (Mueller et al., 2014; Di and Biswal, 2015) and the spatial distribution of functional networks, valid ICs were screened, which belonged to the auditory network (AN), dorsal attention network (DAN), right frontoparietal network (RFPN), left frontoparietal network (LFPN), somatomotor network (SMN), medial visual network (mVN), lateral visual network (pVN), and default mode network (DMN), respectively.

#### 3.1.2 Cluster analysis

First, the Temporal dFNC module in the GIFT software was employed to acquire the dFNC of each patient using the sliding window method. Using the following settings: window size = 30 TRs, TRs = 1.5 s, and step size = 1 TR, a total of 260 windows were obtained for each patient. Then, the k-means clustering algorithm was applied to calculate the Manhattan distance between each window, and clustering was performed on all windows with the maximum number of iterations set to 500 and the number of repetitions to 150. Finally, based on the elbow method, the number of clusters (*k* = 4) was obtained, which implies that dFNC was most optimal when the clustering state was 4.

#### 3.1.3 Calculation of dFNC metrics

The metrics of dFNC results were calculated using MATLAB: (1) fraction time (FT): the percentage of the total number of windows spent by each patient in a given state; (2) mean dwell time (MDT): the average time spent by each patient in a given state; and (3) the number of transitions (NT): the number of times each patient transitions between states.

### 3.2 Quantitative analysis of CSVD imaging features

The CSVD imaging features of each patient were quantified using the CSVD analysis panel of the uAI Discover system (United Imaging Healthcare Co., Ltd., China)^1^ to obtain the WMH volume, number of lacunes, total volume of lacunes, volume of largest lacunar focus, number of PVSs, number of CMBs, total volume of CMBs, volume of largest CMB focus, and bilateral medial temporal lobe atrophy (MTA) scores from each patient. Among which, WMH can be divided into the following four categories: (1) juxtaventricular WMH (≤3 mm from the ventricular surface); (2) periventricular WMH (3–13 mm from the ventricular surface); deep WMH (between periventricular WMH and juxtacortical WMH); and (4) juxtacortical WMH (≤4 mm from the corticomedullary junction).

### 3.3 Statistical analysis

Comparisons between the dFNC groups were performed using the Stats module of the GIFT software package, and the remaining statistical analyses were performed using SPSS software (v27.0.1, United States). General information and dFNC metrics: First, the normality of age, education level, and dFNC metrics were tested for each group by useing Kolmogorov–Smirnov test. The normally-distributed measurement data were expressed as mean ± standard deviation, and subjected to independent samples t-test. Non-normally-distributed measurement data were expressed as median and interquartile range, and subjected to non-parametric tests for between-group comparisons. Categorical variables were subjected to a chi-squared test. Differences with *p* < 0.05 were considered statistically significant.

Between-group comparisons of dFNC: Statistical analyses were performed using the Stats module of the GIFT software package. Two-sample t-tests were performed to compare the dFNC differences for each state between the matched and mismatched groups. *p* < 0.05 was considered statistically significant (FDR corrected).

Correlation analysis between dFNC metrics and cognitive scales: Using the SPSS software, Pearson correlation coefficients were calculated between dFNC metrics with significant between-group differences and scores of the cognitive scales, this was done to examine the correlation between the dFNC metrics and cognitive scales. The results were visualized using scatter plots.

## 4 Results

### 4.1 General information

The 149 CSVD patients included 20 cases (13.4%) with “Type I mismatch,” 51 cases (34.2%) with “Type I match,” 38 cases (25.5%) with “Type II mismatches,” and 40 cases (26.9%) with “Type II match.” The general information, cognitive scale scores, and MRI total Fazekas scores for each group are shown in Table 1. The median Fazekas score was 3 in the mild WMH group and 5 in the severe WMH group (*p* < 0.05). There were significant differences in the scores of all cognitive scales between the groups with and without cognitive impairment (*p* < 0.05). No significant difference was observed in the gender, age, and years of education of patients in the “Type I mismatch” and “Type I match” groups; No significant difference was observed in the gender and age of the “Type II match” and “Type II mismatch” groups, but "Type II mismatch” group had more years of education than “Type II match” group (*p* = 0.005).

**Table 1:** General information of the patients, cognitive scale scores, and MRI total Fazekas score.

	“Type I match”	“Type I mismatch”	*P*-value	“Type II mismatch”	“Type II match”	*P*-value
n(%)	51 (34.2%)	20 (13.4%)		38 (25.5%)	40 (26.9%)	
Gender (male/female)	17/34	9/11	0.359	18/20	18/22	0.834
Age (years)	68 (64–72)^b^	71.5 (66.5–74)^b^	0.068	66.61 (5.09)^a^	68.73 (4.31)^a^	0.051
Years of education	9 (8–9)^b^	8 (6–9)^b^	0.113	9 (8–11)^b^	8 (6–9)^b^	0.005*
MMSE	29 (28–30)^b^	28 (26–29)_b_	0.007*	29 (28–29)^b^	27 (26–28)^b^	<0.001*
MOCA	28 (27–29)^b^	25 (24–26)^b^	<0.001*	28 (27–29)^b^	23.5 (20.25–25.75)^b^	<0.001*
AVLT immediate memory	6.35 (1.29)^a^	4.75 (1.73)^a^	<0.001*	5.9905 (1.57)^a^	4.4195 (1.21)^a^	<0.001*
AVLT delayed memory	5.94 (2.46)^a^	4.05 (2.37)^a^	0.004*	5.5 (4–7)^b^	4 (2–5)^b^	<0.001*
TMT-A	64 (50–79)^b^	102 (79–131)^b^	<0.001*	60.5 (49.25–75.5)^b^	97 (77–132.5)^b^	<0.001*
TMT-B	149 (129–185)^b^	265 (241–365)^b^	<0.001*	150.5 (130.25–182.75)^b^	257.5 (229–341)^b^	<0.001*
Stroop-A	30 (27–34)^b^	37 (30–43)^b^	0.002*	27.5 (24.75–32.25)^b^	38.5 (32–50.5)^b^	<0.001*
Stroop-B	48 (38–58)^b^	67 (59–75)^b^	<0.001*	43 (37.5–50.75)^b^	67 (56–78.75)^b^	<0.001*
Stroop-C	92 (79–105)^b^	146 (113–177)^b^	<0.001*	82 (72–93.25)^b^	147 (109.5–191)^b^	<0.001*
CDT	9 (8–10)^b^	8 (7–9)^b^	0.013*	9 (8–10)^b^	8.5 (8–9)^b^	<0.001*
Total Fazekas score	3 (3–3)^b^	3 (3–3)^b^	0.545	5 (4–6)^b^	5 (4–6)^b^	0.667

^a^Normally distributed data, expressed as mean (standard deviation), and analyzed using chi-squared test.^b^Non-normally distributed data, expressed as median (upper quartile–lower quartile), and analyzed using non-parametric test.*p < 0.05 indicates the difference was statistically significant.

### 4.2 ICA and IC selection

**Figure 1:**
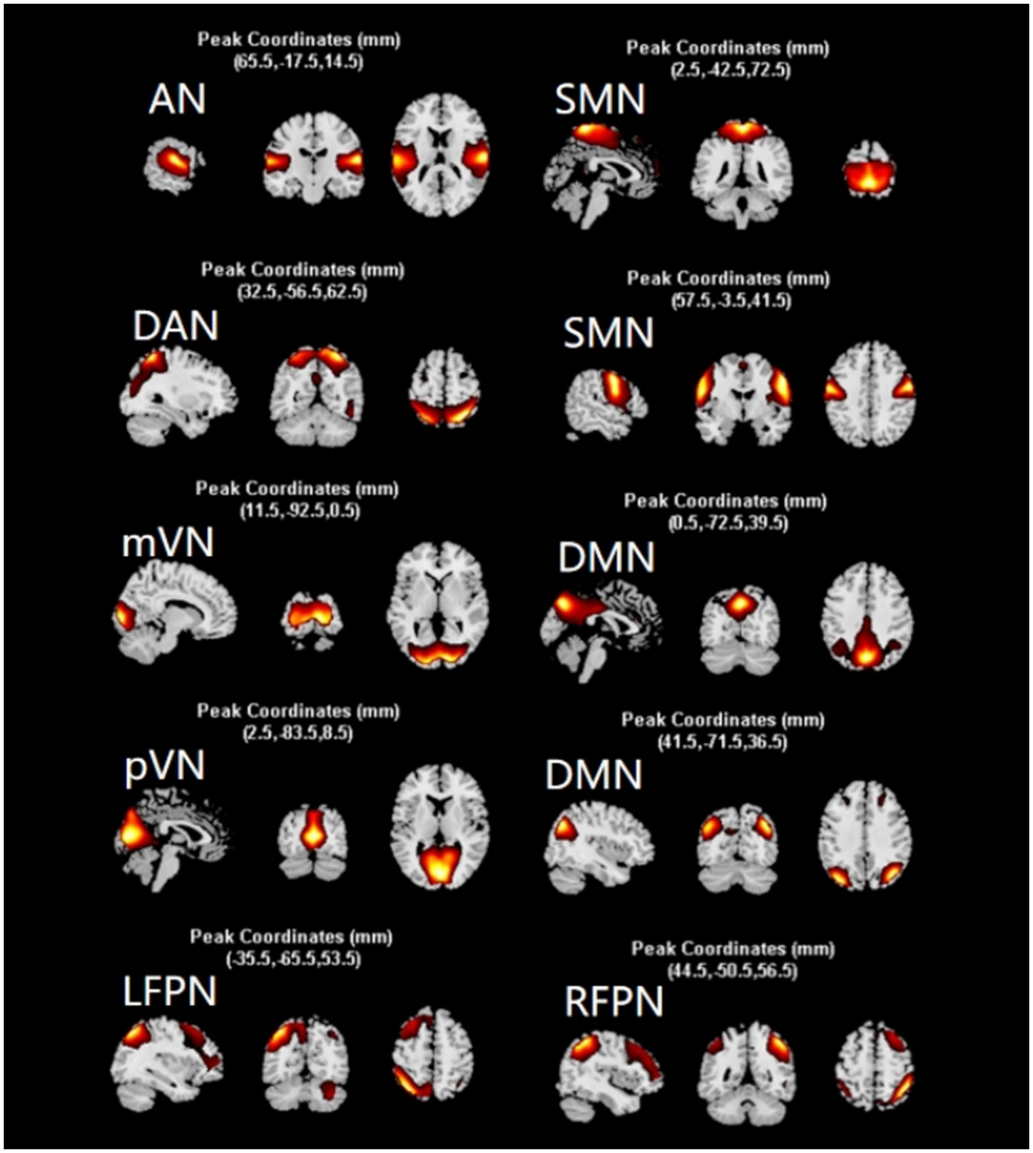
Screening results of independent components. After screening, 10 independent components were obtained, which constituted 8 networks of AN, DAN, SMN, mVN, pVN, DMN, LFPN, and RFPN. network. The diagram shows the distribution of each network. AN, auditory network; DAN, dorsal attention network; RFPN, right frontoparietal network; LFPN, left frontoparietal network; SMN, somatomotor network; mVN, medial visual network; pVN, lateral visual network; DMN, default mode network.

Evaluation using the GIFT software package^2^ yielded 35 ICs. Based on the principle of highest template correlation coefficient and with reference to the spatial distribution of functional networks in the relevant literature (Mueller et al., 2014; Di and Biswal, 2015), 10 valid ICs were selected involving 8 Resting-State Networks, namely AN (IC2), DAN (IC27), RFPN (IC25), LFPN (IC11), SMN (IC7, IC1), mVN (IC4), pVN (IC12), and DMN (IC29, IC21) (Figure 1).

### 4.3 Cluster analysis

**Figure 2:**
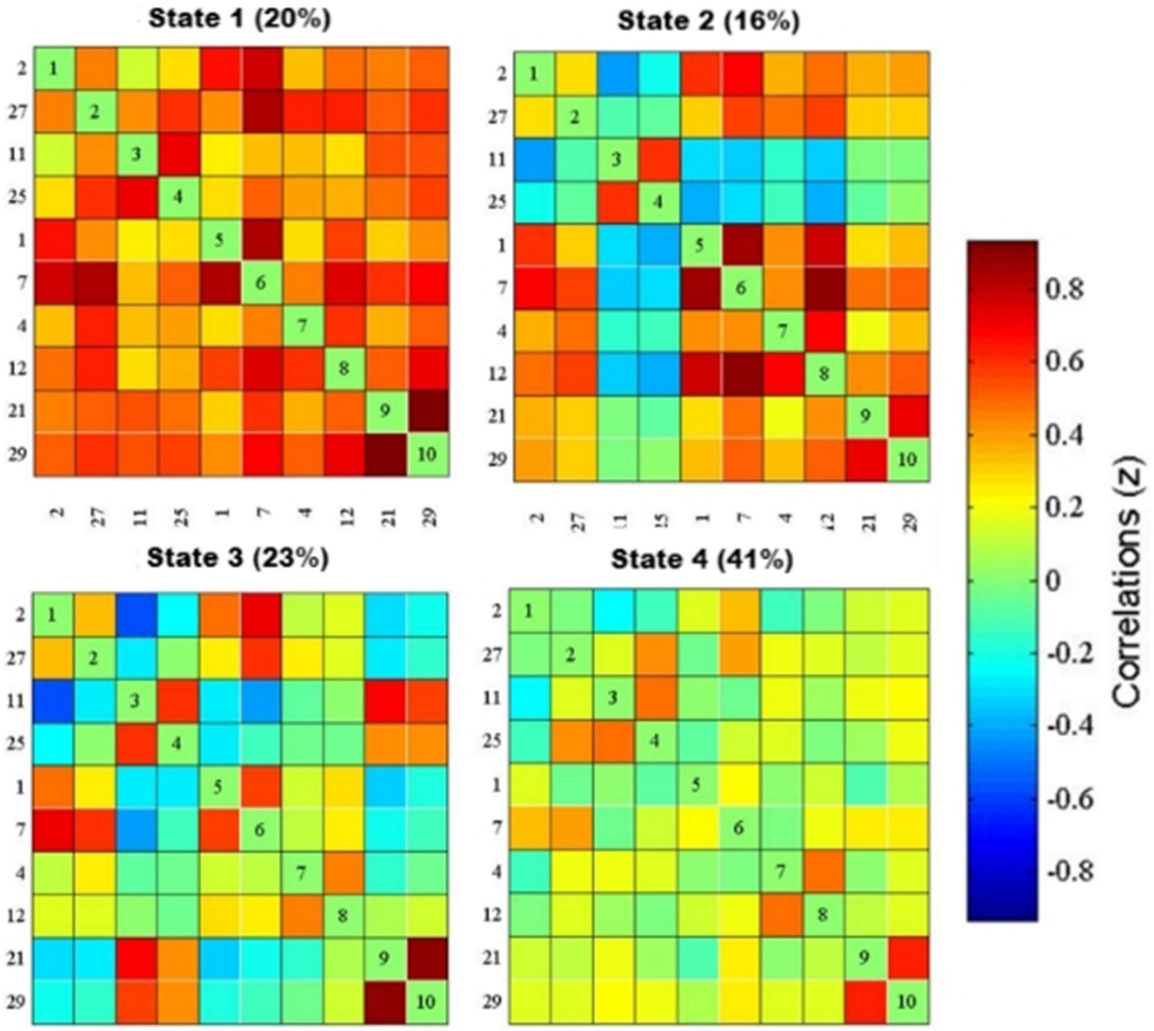
dFNC matrix of the four states and their percentages. The functional network connection matrix constructed by IC2, IC27, ICII, IC25, ICI, IC7, IC4, IC12, IC21, and IC29 independent components is clustered to obtain four states, red represents positive connection, blue represents negative connection, and the darker the color, the stronger the correlation: In state 1 (20%), the functional network connection is strong; in state 3 (23%) and state 4 (41%), the functional network connection is weak; in state 2 (16%), the functional network connection is intermediate between the strong connection and the weak connection.

**Figure 3:**
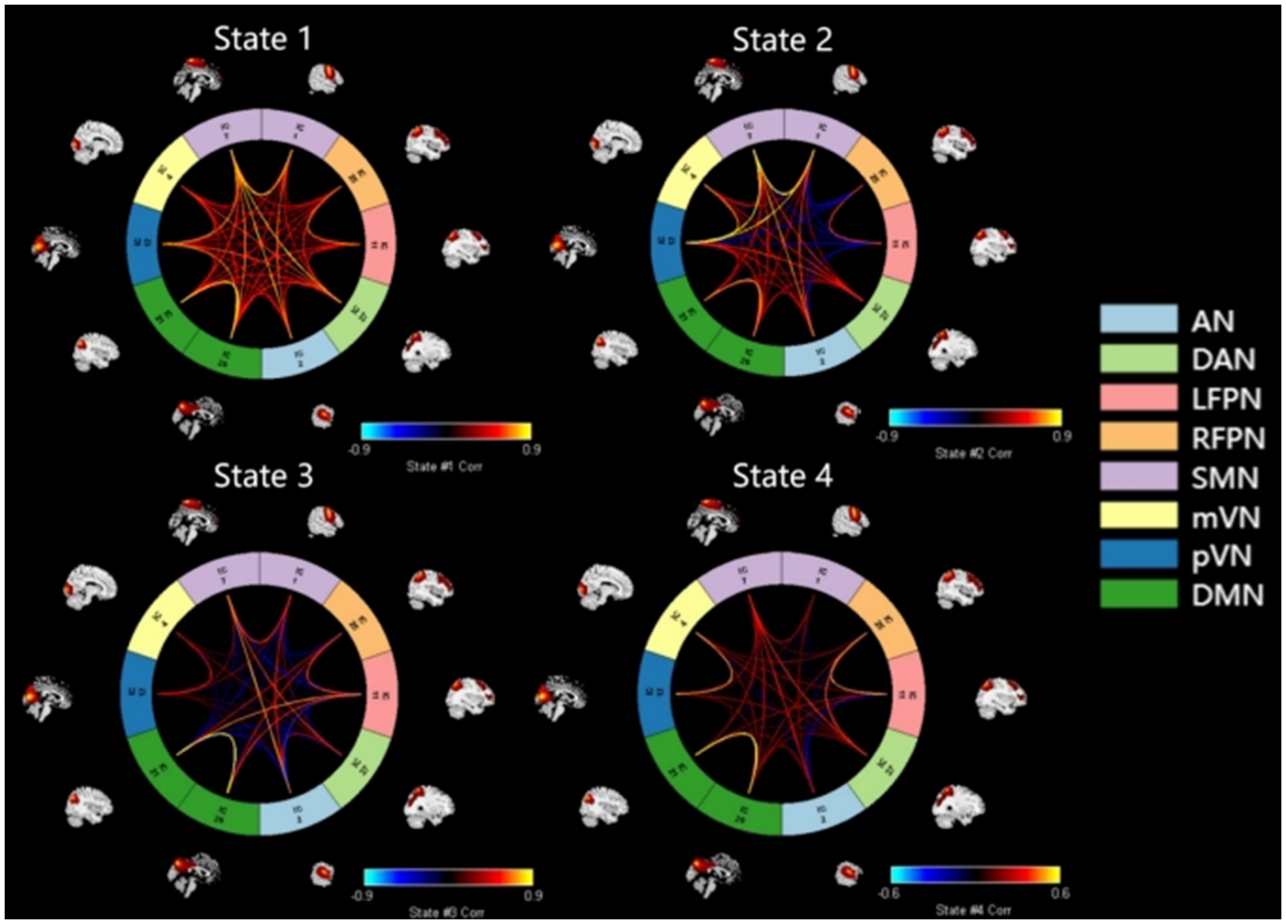
Circular plots of dFNC for states 1–4. Functional network connection circle diagram constructed by 10 independent components of IQ, IC27, ICII, IC25, ICI, IC7, IC4, IC12, IC21, IC29 and 8 networks of AN, DAN, SMN, mVN, pVN, DMN, LFPN, RFPN, red represents forward connection. The brighter the color, the stronger the correlation, the darker the color, the weaker the correlation: the functional network connection in state 1 is strong, the functional network connection in state 3 and state 4 is weak, the functional network connection is weak, and the state 2 is between strong and weak, the intermediate state. AN, auditory network; DAN, dorsal attention network; RFPN, right frontoparietal network; LFPN, left frontoparietal network; SMN, somatomotor network; mVN, medial visual network; pVN, lateral visual network; DMN, default mode network.

K-means cluster analysis was performed based on the optimal number of clusters, which yielded a total of four states. Among them, State 4 (41%) was the most common, followed by State 3 (23%), State 1 (20%), and State 2 (16%) (Figure 2). For more intuitive visualization, the matrices were transformed into circular plots (Figure 3). Differing FNC patterns and strengths between different states: In State 1, almost all inter- and intra-network connectivities were strong and positive, especially the inter-network connectivity between DAN and SMN, and between DMN and SMN, with AN and SMN showing slightly strong inter-network connectivity. State 4 is a weakly connected state with significantly lower strengths of inter- and intra-network connectivity across all networks compared to State 1. States 2 and 3 showed the coexistence of positive and negative connectivities, as well as strong and weak connectivities. Hence, these are intermediate states between States 1 and 4. Among these States, State 2 showed stronger connectivity between SMN and pVN, and within SMN, while State 3 showed stronger connectivity within DMN.

### 4.4 Between-group comparisons of dFNC

Between-group comparisons of the FNC matrices for each dFNC state were performed using the Stats module of the GIFT software package.

**Figure 4:**
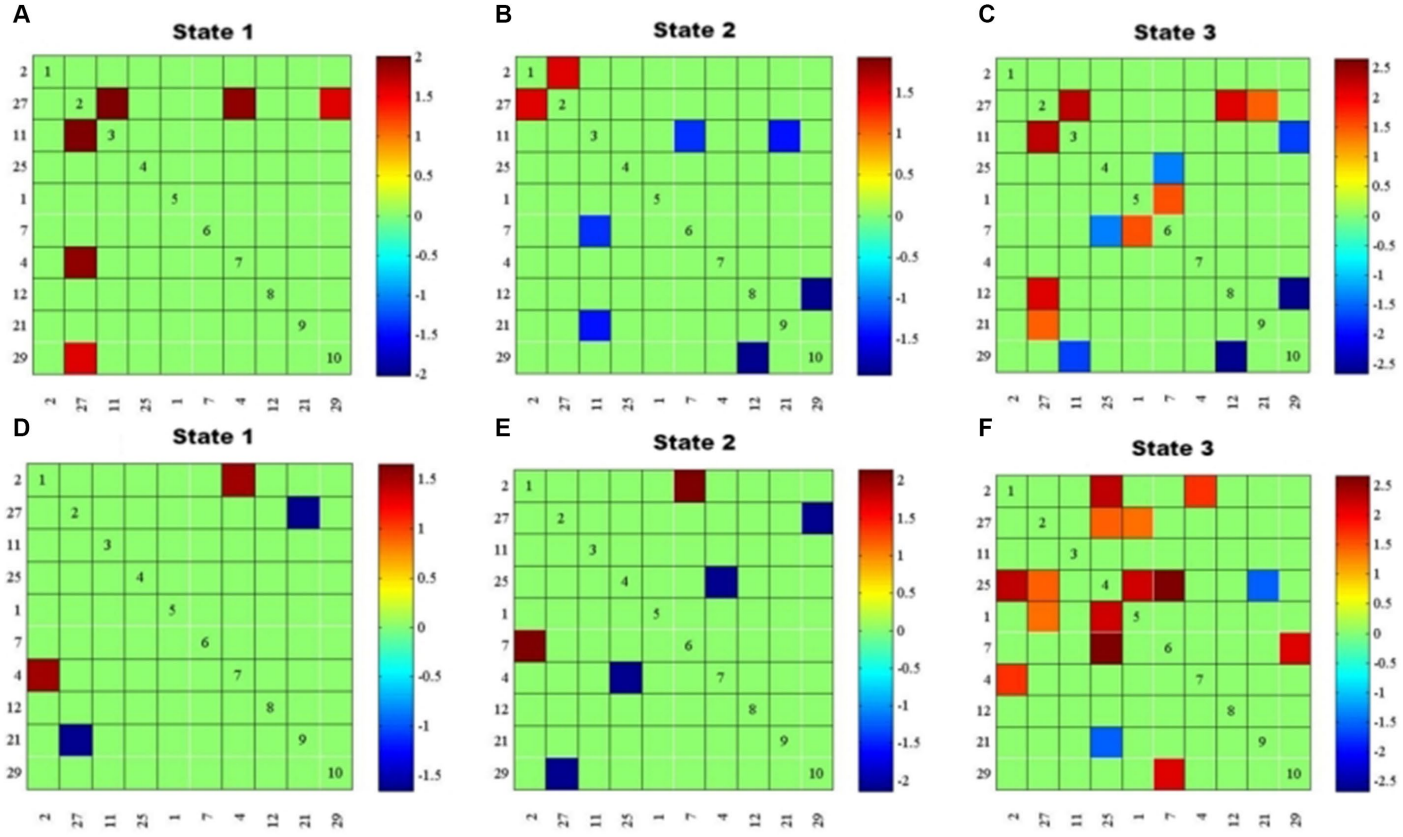
Results of dFNC between-group comparisons by state (*P* < O.05, FDR corrected). **(A–C)** FNC differences between “Type I match” and “Type I mismatch” groups for states 1–3. **(D–F)** FNC differences between “Type Il match” and “Type I l mismatch” groups for states 1–3. Red represents positive connections, blue represents negative connections, and the darker the color, the stronger the correlation.

In State 1, compared with the “Type I match” group, the “Type I mismatch” group showed weaker FNC of DAN with DMN, mVN, and LFPN, respectively. In State 2, the mismatch group showed stronger FNC between DMN and pVN, and of LFPN with SMN and DMN, respectively, as well as weaker FNC between DAN and AN. In State 3, the mismatch group showed stronger FNC of DMN with pVN and LFPN, respectively, and between SMN and RFPN; as well as weaker FNC of DAN with LFPN, pVN, and DMN, respectively, and within SMN (Figures 4A–C).

Compared with the “Type II match” group, the “Type II mismatch” group showed stronger FNC between AN and pVN, and weaker FNC between DMN and DAN in State 1. In State 2, the cognitive impairment group showed stronger FNC between AN and SMN; as well as weaker FNC between DMN and DAN, and between mVN and RFPN. In State 3, the cognitive impairment group showed stronger FNC of AN with mVN and RFPN, respectively, and of DAN with SMN and RFPN, respectively, between RFPN and SMN, and between DMN and SMN; as well as weaker FNC between DMN and RFPN (Figures 4D–F).

### 4.5 Between-group comparisons of higher-order dFNC metrics

Using MATLAB, higher-order metrics of the four states were extracted, including FT, MDT, and NT, and statistical analysis was performed using SPSS 27. NT conformed to a normal distribution (S–W test, *p* > 0.05), and was analyzed using the two-sample t-test. FT and MDT did not conform to a normal distribution (S–W test, *p* < 0.05), and were analyzed using a non-parametric test (Mann–Whitney U test) (see Table 2).

**Table 2:** dFNC metrics by group.

	“Type I match”	“Type I mismatch”	*P*-value	“Type II mismatch”	“Type II match”	*P*-value
FT1	0.112 (0.019–0.255)^b^	0.129 (0.006–0.274)^b^	0.837	0.112 (0.019–0.255)^b^	0.129 (0.006–0.274)^b^	0.017*
FT2	0.151 (0.039–0.251)^b^	0.035 (0.000–0.142)^b^	0.009*	0.151 (0.039–0.251)^b^	0.035 (0–0.142)^b^	0.372
FT3	0.139 (0.027–0.297)^b^	0.199 (0.027–0.608)^b^	0.185	0.139 (0.027–0.297)^b^	0.199 (0.027–0.608)^b^	0.775
FT4	0.431 (0.253)^a^	0.443 (0.282)^a^	0.860	0.402 (0.220–0.664)^b^	0.363 (0.222–0.680)^b^	0.04*
MDT1	14.33 (5.00–24.00)^b^	12.45 (1.50–22.56)^b^	0.626	14.33 (5.00–24.00)^b^	12.45 (1.50–22.56)^b^	0.162
MDT2	13.00 (5.67–22.00)^b^	6.00 (0.00–11.38)^b^	0.004*	13.00 (5.67–22.00)^b^	6.00 (0.00–11.38)^b^	0.234
MDT3	10.75 (4.60–24.83)^b^	17.25 (5.25–26.77)^b^	0.522	10.75 (4.60–24.83)^b^	17.25 (5.25–26.77)^b^	0.666
MDT4	18.00 (10.63–36.60)^b^	14.50 (9.69–36.46)^b^	0.614	18.00 (10.63–36.60)^b^	14.50 (9.69–36.46)^b^	0.078
NT	11.00 (8.00–15.00)^b^	11.50 (9.25–15.50)^b^	0.696	12.63 (0.89)^a^	12.03 (0.87)^a^	0.627

^a^Normally distributed data, expressed as mean (standard deviation), and analyzed using chi-squared test.^b^Non-normally distributed data, expressed as median (upper quartile–lower quartile), and analyzed using non-parametric test.*P < 0.05 indicates the difference was statistically significant.WMH, white matter hyperintensity; FT, fraction time; MDT, mean dwell time; NT, number of transitions.

In the Type I patients, the State 2 FT (FT2) and MDT (MDT2) differed significantly between the match and mismatch groups (*p* < 0.05), whereas the differences in the remaining dFNC metrics were not statistically significant (*p* > 0.05).

**Figure 5:**
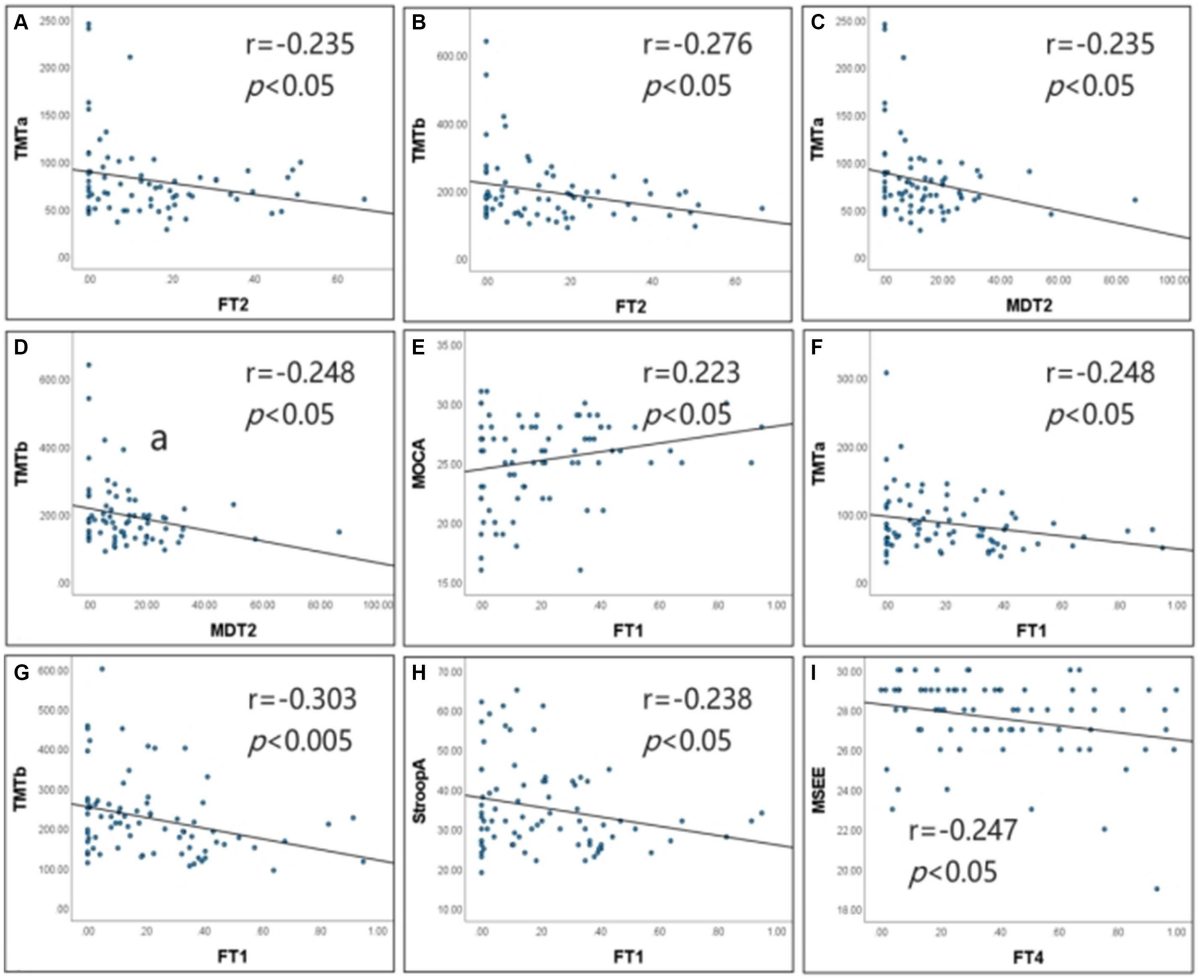
Correlation of dFNC metrics with cognitive scales. **(A–D)** Show the results of the correlation analysis for Type I patients. **(E–I)** Show the results of the correlation analysis for Type Il patients. FTI, fraction time of State 1; FT2, fraction time of State 2; FT4, fraction time of State 4; MDT2, mean dwell time of State 2; TMTa, Digital Connection Test a; TMTb, Digital connection test b; StroopA, Stroop color word test A; MoCA, Montreal Cognitive Assessment; MMSE, Simple mental state Checklist. In type I patients, FT2 was negatively correlated with MTT-A and the correlation coefficient was −0.235 (*p* < 0.05). FT2 was negatively correlated with MTT-B, and the correlation coefficient was −0.276 (*p* < 0.05). MDT2 was negatively correlated with MTT-A, and the correlation coefficient was −0.235 (*p* < 0.05). MDT2 was negatively correlated with MTT-B, and the correlation coefficient was −0.248 (*p* < 0.05). ln type Il patients, FTI was negatively correlated with and the correlation coefficient was −0.248 (*p* < 0.05). FTI was negatively correlated with MTT-B, and the correlation coefficient was −0.303 (*p* < 0.01). FTI was negatively correlated with StroopA, and the correlation coefficient was −0.238 (*p* < 0.05). FTI was positively correlated with MoCA, and the correlation coefficient was 0.223 (*p* < 0.05). FT4 Was negatively correlated with MMSE, and the correlation coefficient was −0.247 (*p* < 0.05).

In the Type II patients, the FT for states 1 and 4 (FT1, FT4) differed significantly between the match and mismatch groups (*p* < 0.05), whereas the differences of the remaining dFNC metrics were not statistically significant (*p* > 0.05).

### 4.6 Correlation analysis of dFNC and its higher-order metrics with cognitive scale scores

Correlation analysis revealed that in the Type I patients, FT2negatively correlated with TMT-A (*r* = −0.235, *p* < 0.05) and TMT-B (*r* = −0.276, *p* < 0.05); MDT2 negatively correlated with TMT-A (*r* = −0.235, *p* < 0.05) and TMT-B (*r* = −0.248, *p* < 0.05); FT2 and MDT2 did not significantly correlate with the remaining cognitive scales. In the Type II patients, FT1 negatively correlated with TMT-A (*r* = −0.248, *p* < 0.05), TMT-B (*r* = −0.303, *p* < 0.01) and Stroop-A (*r* = −0.238, *p* < 0.05), and it positively correlated with MoCA (*r* = 0.223, *p* < 0.05) and CDT (*r* = 0.248, *p* < 0.05); FT4 negatively correlated with MMSE (*r* = −0.247, *p* < 0.05); FT1 and FT4 did not significantly correlate with the remaining cognitive scales (Figure 5).

### 4.7 Quantitative analysis of CSVD imaging features

Quantification of CSVD imaging features showed that the “Type I mismatch” group had a significantly higher number of PVSs (*p* < 0.05) and higher bilateral MTA scores (*p* < 0.05) than the “Type I match” group; the “Type II mismatch” group had a significantly lower left MTA score than the “Type II match” group (*p* < 0.05) (Table 3).

**Table 3:** Quantification of CSVD imaging features.

	“Type I match”	“Type I mismatch”	*P*-value	“Type II mismatch”	“Type II match”	*P*-value
Juxtaventricular WMH volume	2.90 (2.18–3.91)^b^	3.66 (2.27–3.97)^b^	*P* > 0.05	5.09 (3.71–6.556)^b^	5.02 (3.47–7.41)^b^	*P* > 0.05
Periventricular WMH volume	0.58 (0.26–1.48)^b^	0.83 (0.58–1.07)^b^	*P* > 0.05	3.2 (1.94–6.92)^b^	4.47 (1.80–7.23)^b^	*p* > 0.05
Deep WMH volume	0.54 (0.14–1.30)^b^	0.18 (0.06–0.61)^b^	*P* > 0.05	2.37 (1.28–4.65)^b^	1.57 (0.77–3.93)^b^	*P* > 0.05
Juxtocortical WMH volume	0.35 (0.18–0.55)^b^	0.31 (0.18–0.71)^b^	*P* > 0.05	0.74 (0.39–1.70)^b^	0.7 (0.44–1.60)^b^	*P* > 0.05
Number of lacunes	0.00 (0.00–0.75)^b^	0.00 (0.00–0.00)^b^	*P* > 0.05	0.00 (0.00–1.00)^b^	0.00 (0.00–1.00)^b^	*P* > 0.05
Total volume of lacunes	0.00 (0.00–4.09)^b^	0.00 (0.00–0.00)^b^	*P* > 0.05	0.00 (0.00–48.60)^b^	0.00 (0.00–61.99)^b^	*P* > 0.05
Volume of largest lacunar focus	0.00 (0.00–4.09)^b^	0.00 (0.00–0.00)^b^	*P* > 0.05	0.00 (0.00–48.60)^b^	0.00 (0.00–61.99)^b^	*P* > 0.05
Number of PVSs	37.39 (17.05)^a^	52.00 (26.17)^a^	*p* = 0.034*	32.50 (22.00–54.00)^b^	31.00 (23.00–53.00)^b^	*P* > 0.05
Number of CMBs	0.00 (0.00–1.00)^b^	0.00 (0.00–1.00)^b^	*P* > 0.05	1.00 (0.00–2.00)^b^	0.00 (0.00–1.50)^b^	*P* > 0.05
Total volume of CMBs	0.00 (0.00–13.56)^b^	0.00 (0.00–49.15)^b^	*P* > 0.05	16.93 (0.00–60.05)^b^	0.00 (0.00–27.12)^b^	*P* > 0.05
Volume of largest CMB focus	0.00 (0.00–12.92)^b^	0.00 (0.00–33.72)^b^	*P* > 0.05	16.93 (0.00–52.81)^b^	0.00 (0.00–25.18)^b^	*P* > 0.05
Left MTA score	0.00 (0.00–1.00)^b^	1.00 (0.00–1.00)^b^	*p* = 0.048*	0.50 (0.00–1.00)^b^	1.00 (0.00–1.00)^b^	*p* = 0.020*
Right MTA score	0.00 (0.00–1.00)^b^	1.00 (0.00–1.00)^b^	*p* = 0.039*	0.00 (0.00–1.00)^b^	1.00 (0.00–1.00)^b^	*P* > 0.05

^a^Normally distributed data, expressed as mean (standard deviation), and analyzed using chi-squared test.^b^Non-normally distributed data, expressed as median (upper quartile–lower quartile), and analyzed using non-parametric test.*P < 0.05 indicates the difference was statistically significant.WMH volume, total volume of lacunes, volume of largest lacunar focus, total volume of CMBs, and volume of largest CMB focus are in mm^3^.WMH, white matter hyperintensity; CMBs, cerebral microbleeds; MTA, medial temporal lobe atrophy.

## 5 Discussion

A subset of patients with CSVD exhibited a mismatch between WMH and cognitive function (Pasi et al., 2016; Wang et al., 2021), but the reason for this observation remains unclear. Currently, brain fMRI is frequently used to explore the underlying pathogenesis of neuropsychiatric disorders (Logothetis, 2008), while quantitative analysis allows for a more objective and detailed characterization of WMH and other imaging features of CSVD. Therefore, in this study, we explored the dFNC differences between the match and mismatch groups using brain fMRI, and we attempted to explain the mismatch phenomenon using quantitative analysis. First, we observed that there were differences between the match and mismatch groups with respect to the dFNC of certain networks and metrics of each state. Furthermore, the differences between Type I and Type II were observed in different networks and metrics, which has not been reported in previous studies. Second, correlation analyses demonstrated that certain dFNC metrics could reflect executive function and information processing speed in CSVD patients, which is consistent with the results of previous studies (Yin et al., 2022; Si et al., 2023). Finally, quantitative analysis showed that there were significant differences in PVSs and bilateral MTA scores between the “Type I match” and “Type I mismatch” groups, whereas the “Type II match” and “Type II mismatch” groups only differed significantly with respect to the left MTA score. PVSs and brain atrophy may be independent risk factors for cognitive impairment (Fan et al., 2021; Sun et al., 2022).

### 5.1 dFNC analysis

In this study, we compared the dFNC differences between the mismatch and match groups, which have not been reported in the literature within this field. However, in a related study, they found that there were differences in some FNC between CSVD patients with or without cognitive impairment, showing both FNC enhancement and attenuation. Compared to CSVD patients without cognitive impairment, those with cognitive impairment exhibited attenuated FNC among AN, DMN, and RFPN, as well as enhanced FNC among DMN, AN, and VN (Pei et al., 2020), which is similar to the results obtained during this study.

Comparisons between the “Type I match” and “Type I mismatch” groups revealed that FNC changes occurred in networks such as DMN and DAN. In particular, DMN is associated with self-awareness, introspection, memory, and cognitive control, while DAN is associated with attention, eye movements, and visual search (Raichle, 2015). DMN and DAN are higher-order networks that are involved in more advanced and complex cognition and information integration in the brain, consisting of areas associated with higher-order cognitive functions such as executive function, decision-making, emotional processing, memory, and language. Higher-order networks go beyond the preliminary processing of sensory input and instead are involved in the analysis, integration, and reasoning of information at a higher level. The connectivity between these functional networks is complex and dynamic. For example, the DAN can work in concert with the VN to direct attention in visual tasks; the DMN works in opposition to attentional networks (e.g., DAN and FPN) such that when one is activated, the other is inhibited; the SMN can work in concert with other networks to coordinate body movements in motor and perceptual tasks (Corbetta and Shulman, 2002; Dosenbach et al., 2008; Rauschecker and Scott, 2009; Biswal et al., 2010; Grefkes and Fink, 2011; Raichle, 2015).

While comparing the “Type II match” and “Type II mismatch” groups, FNC changes were observed in networks such as the AN and mVN, of which, the AN includes the auditory cortex, and is responsible for processing sound and auditory information (Rauschecker and Scott, 2009), while the VN is responsible for processing visual information, including the perception of shape, color, and motion (Biswal et al., 2010). The AN and VN are primary networks, encompassing brain regions that process basic sensory and perceptual information. These networks include the primary sensory cortices, which are responsible for the reception and initial processing of information from the sensory organs. They are generally involved in the early stages of information processing, and they play a pivotal role in the simple perception of external stimuli.

The dFNC differences between the two types of mismatch groups and match groups were observed in different networks, suggesting that different mechanisms may be involved in giving rise to the two types of mismatches. This is also reflected in the dynamic metrics of dFNC: Compared with the “Type I match” group, the “Type I mismatch” group had a lower FT2 and shorter MDT2. In other words, the “Type I mismatch” group showed a lower FT and shorter MDT for the intermediate states, and hence exhibited more polarized dynamics within their brain functional networks. This may be one of the causes of the early onset of their cognitive impairment. Compared to the “Type II match” group, the “Type II mismatch” group had a lower FT1 and higher FT4, that is, patients had a lower FT for the strongly connected state and higher FT for the weakly connected state.

### 5.2 Correlation between dFNC metrics and cognition

Based on the results of our correlation analysis, FT1 positively correlated with the overall cognitive function, and FT4 negatively correlated with overall cognitive function, which is similar to the results from a previous dFNC study on CSVD with or without cognitive impairment (Yin et al., 2022). Furthermore, in the Type I patients, FT2 negatively correlated with TMT-A and TMT-B, and MDT2 negatively correlated with TMT-A and TMT-B as FT2 and MDT2 increased, which implies that patients with higher FT2 and MDT2 had better executive function and faster information processing speed. In the Type II patients, FT1 negatively correlated with TMT-A, TMT-B, and Stroop-A, which implies that patients with higher FT1 had better executive function and faster information processing speed. Therefore, the dynamic metrics of the intermediate states in Type I patients and the strongly connected state in Type II patients correlated with the executive function and information processing speed in patients with CSVD.

### 5.3 Quantitative analysis of CSVD imaging features

Since the dFNC differences between the mismatch and match groups were different for the Type I and Type II patients, we speculated that the mechanism that gave rise to the occurrence of “Type I mismatch” was different from that of the “Type II mismatch.” To explain the mechanisms underlying the two types of mismatches, we performed quantitative analyses to compare information such as WMH, lacunes, PVSs, CMBs, and MTA scores between the two sets of mismatch and match groups.

The “Type I mismatch” group had a significantly higher number of PVSs and higher bilateral MTA scores. Therefore, the primary mechanism of the “Type I mismatch” may involve cognitive impairment mediated by an increase in PVSs (Yang et al., 2022) and, to a lesser extent, bilateral brain atrophy. PVSs refer to the spaces around small arteries, capillaries, and small veins (Wardlaw et al., 2020). They have been shown to play an important role in the brain lymphatic system (Yu et al., 2022) and are closely associated with WMH and cognitive impairment (Wang et al., 2021). Research has found that the location and characteristics of perivascular spaces in the basal ganglia and centrum semiovale differ, possibly indicating different pathophysiological significances (Doubal et al., 2010). Therefore, the spatial distribution of perivascular spaces is also related to cognitive function. Additionally, studies have confirmed that CSVD, Alzheimer’s disease, and mixed forms have different etiological distributions (Ramusino et al., 2022).

The left MTA score was lower in the “Type II mismatch” group, which implies that the occurrence of the “Type II mismatch” may be because atrophy had not yet occurred in the left hemisphere. It has been demonstrated in previous studies that MTA scores are associated with cognitive impairment in patients with CSVD (Sun et al., 2022). In addition, the educational levels between the “Type II mismatch” and “Type II match” groups were different, which may be related to the cognitive reserve reported in previous studies (Pinter et al., 2015).

### 5.4 Study limitations

First, this was a cross-sectional study, which did not include the follow-up of CSVD patients for longitudinal research to validate the current findings. Second, we observed some correlation between the dFNC metrics and cognition, but the correlation was weak, which may have been related to the small sample size. Third, slight differences in the dFNC and its metrics were observed between the match and mismatch groups, which were insufficient to provide a mechanistic explanation (e.g., neural transmission connectivity pathways). Thus, these findings need to be supplemented by relevant structural network studies and basic experiments in the future. Finally, the quantitative analysis in this study only provided a superficial explanation of the mismatch phenomenon, and further in-depth studies are needed to divide patients by CSVD overall load score.

## 6 Conclusion

Using dFNC analysis, we were able to identify significant differences in the functional networks involved in the Type I and Type II mismatch phenomena, suggesting that different mechanisms were responsible for these two types of mismatch: Type I affected higher-order networks and may be related to the number of PVSs, whereas Type II affected the primary networks and may be related to brain atrophy and cognitive reserve. Furthermore, the dFNC metrics in CSVD patients correlated with the executive function and information processing speed among the various cognitive functions.

## Data availability statement

The raw data supporting the conclusions of this article will be made available by the authors, without undue reservation.

## Author contributions

SZ: Conceptualization, Data curation, Formal analysis, Methodology, Visualization, Writing – original draft. LM: Data curation, Formal analysis, Methodology, Writing – review & editing. HM: Data curation, Methodology, Project administration, Writing – review & editing. YS: Data curation, Project administration, Writing – review & editing. MX: Data curation, Writing – review & editing. QG: Data curation, Writing – review & editing. CK: Data curation, Writing – review & editing. ML: Data curation, Writing – review & editing. YD: Data curation, Writing – review & editing. YJ: Data curation, Writing – review & editing. XH: Project administration, Supervision, Writing – review & editing. WF: Project administration, Supervision, Writing – review & editing. XF: Project administration, Supervision, Writing – review & editing.

## Ethics statement

The studies involving humans were approved by the Research Ethics Committee of Wuxi People’s Hospital. The studies were conducted in accordance with the local legislation and institutional requirements. The participants provided their written informed consent to participate in this study.

## Footnotes

^1^www.uii-ai.com

^2^trendscenter.org
